# Energy-Balanced Cluster-Routing Protocol Based on Particle Swarm Optimization with Five Mutation Operators for Wireless Sensor Networks

**DOI:** 10.3390/s20247217

**Published:** 2020-12-16

**Authors:** Yamin Han, Heejung Byun, Liangliang Zhang

**Affiliations:** 1 Department of Computer Science, The University of Suwon, Hwaseong 18323, Korea; amyhan@suwon.ac.kr (Y.H.); leonzhang@suwon.ac.kr (L.Z.); 2 Department of Information and Technology, The University of Suwon, Hwaseong 18323, Korea

**Keywords:** wireless sensor networks, cluster, energy balance, particle swarm optimization, mutation operators

## Abstract

Prolonging the network lifetime is one of the fundamental requirements in wireless sensor networks (WSNs). Sensor node clustering is a very popular energy conservation strategy in WSNs, allowing to achieve energy efficiency, low latency, and scalability. According to this strategy, sensor nodes are grouped into several clusters, and one sensor node in each cluster is assigned to be a cluster head (CH). The responsibility of each CH is to aggregate data from the other sensor nodes within its cluster and send these data to the sink. However, the distribution of sensor nodes in the sensing region is often non-uniform, which may lead to an unbalanced number of sensor nodes between clusters and thus unbalanced energy consumption between CHs. This, in turn, may result in a reduced network lifetime. Furthermore, a different number of clusters lead to a different quality of service of a WSN system. To address the problems of unbalanced number of sensor nodes between clusters and selecting an optimal number of clusters, this study proposes an energy-balanced cluster-routing protocol (EBCRP) based on particle swarm optimization (PSO) with five mutation operators for WSNs. The five mutation operators are specially proposed to improve the performance of PSO in optimizing sensor node clustering. A rotation CH selection scheme based on the highest residual energy is used to dynamically select a CH for each cluster in each round. Simulation results show that the proposed EBCRP method performs well in balancing energy consumption and prolonging the network lifetime.

## 1. Introduction

Wireless sensor networks (WSNs) compose a large number of sensor nodes, which are scattered in the sensing region, and one or more sink nodes [1]. Generally, sensor nodes are low-cost, energy-limited, static, and randomly deployed in the sensing region. They are used to obtain such data as temperature, humidity, and air pressure in a physical environment. Communication between sensor nodes or between sensor nodes and sink is realized through wireless signals. Sensor nodes can send data obtained from an environment to a sink in one hop or multiple hops. WSNs have been widely used for environment detection, surveillance and defense reconnaissance, and medical monitoring [2,3,4].

Prolonging the network lifetime is one of the fundamental requirements in WSNs. The lifetime of a network is mainly affected by energy consumption by sensor nodes. A significant amount of the energy consumed by sensor nodes is used for data transmission [5,6,7]. However, replacing or charging batteries of sensor nodes is not possible in some environments such as mines and battlefields [8]. Therefore, it is crucial to make an effective strategy for transmitting data sensed by sensor nodes to the sink node to prolong the network lifetime.

**Figure 1:**
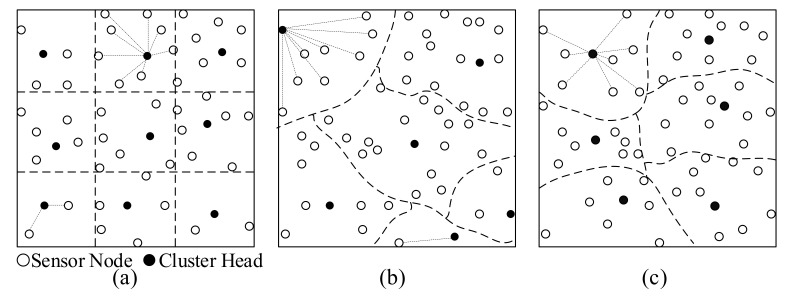
Simplified diagram of sensor node clustering: (**a**) grid-based clustering method, (**b**) low-energy adaptive clustering hierarchy (LEACH)-based clustering method, and (**c**) adaptive sensor node clustering method for determining the number of clusters and grouping the number of sensor nodes into clusters evenly.

Sensor node clustering is one of the effective strategies for prolonging the network lifetime [9,10,11,12]. According to this strategy, sensor nodes are divided into several groups, referred to as clusters. For each cluster, one sensor node is selected as a cluster head (CH), while the remaining sensor nodes are considered as cluster members. These cluster members send their sensed data to their CH. Then, the CH transmits the data aggregated from all the cluster members to the sink in one hop or multiple hops. The sensor node clustering strategy has many benefits such as energy efficiency, low latency, and scalability [13,14]. Many clustering methods have been proposed to prolong the network lifetime. One of the methods divides the sensing region equally into many grids (subregions) [10,11,12], with sensor nodes in each grid being regarded as a cluster (Figure 1a). Another popular clustering method called low-energy adaptive clustering hierarchy (LEACH) can prolong the network lifetime [9]. In LEACH, CHs are selected from sensor nodes based on a random probability, with the remaining sensor nodes being assigned to their corresponding nearest CH (Figure 1b). Furthermore, in order to improve the performance of clustering sensor nodes, scholars have improved the LEACH-based methods, for example, modifying CH selection algorithms [15,16,17], energy aware algorithms [18,19,20], and optimization in CH selection [21,22,23].

However, the clustering of sensor nodes still faces many challenges. Generally, sensor nodes are randomly scattered in the sensing region. Grid-based clustering or assigning sensor nodes to their corresponding nearest randomly selected CH may cause an imbalance in the number of sensor nodes between clusters, resulting in different energy consumption by CHs. A CH within a cluster containing a large number of sensor nodes would consume a lot of energy and be exhausted quickly. Furthermore, the number of clusters affects the performance of WSNs. On the one hand, a small number of clusters would increase the energy consumption of each CH when gathering data from sensor nodes within its cluster and sensor nodes far away from the CH when sending data to the CH. On the other hand, a large number of clusters would increase the traffic burden in the network. Therefore, a crucial task for increasing the network lifetime is to adaptively determine the number of clusters and group sensor nodes into clusters evenly according to their distribution. This is important to improve the performance of WSN such as the network lifetime.

Recently, computational intelligence has been widely used in WSNs to provide solutions for some problems such as nodes deployment, task allocation, path planning, and movement control [24,25,26]. Computational intelligence has the ability to deal with imprecise information and find approximate yet good-enough solutions to these problems [27]. Computational intelligence methods are often inspired by biological processes. As one of the technologies in computational intelligence, evolutionary computation [28] imitates the process of natural evolution to provide a near-optimal solution for an optimization problem. The advantages of evolutionary computation are self-organization, self-learning, and parallel search for the global optimal solution. Among many evolutionary algorithms, the particle swarm optimization algorithm (PSO) can find the optimal solution to a problem at a higher velocity by considering previous global and local best experiences of the entire population.

This study proposes an energy-balanced cluster-routing protocol (EBCRP) using PSO with five mutation operators for WSN that groups sensor nodes into clusters evenly. In the step of EBCRP, the number of clusters and their centroids is optimized (Figure 1c), in which some sensor nodes are selected as the centroids of clusters and other sensor nodes are assigned to the nearest centroid. The average distance between sensor nodes belonging to one cluster and the centroids of this cluster, as well as balancing the number of sensor nodes between clusters are used as optimization objectives. Five mutation operators are specially designed to improve the performance of PSO in optimizing the clustering of sensor nodes. According to the clustering results of sensor nodes, in each round, the sensor node with the highest residual energy in each cluster has the priority to be selected as a CH to aggregate data from other sensor nodes in the same cluster. Then, these CHs are responsible for sending aggregated data to the sink. The proposed schemes for balanced clustering scheme and rotation CH selection based on the highest residual energy are helpful in balancing the energy consumption of sensor nodes and thus prolonging the network lifetime. The main contributions of this work are the following:

- An energy-balanced cluster-routing protocol for WSNs is proposed to balance the energy consumption of sensor nodes and prolong the network lifetime.
- An adaptive sensor node clustering scheme based on PSO is proposed to determine the number of clusters and group sensor nodes into clusters evenly.
- Five mutation operators are specially proposed to improve the performance of PSO in optimizing the clustering of sensor nodes.

The rest of the paper is organized as follows. Section 2 introduces related works on clustering sensor nodes and prolonging the network lifetime. The considered network model is described in Section 3. Section 4 outlines the proposed EBCRP method. Simulations and their results are represented in Section 5. Section 6 concludes the study and suggests directions for future work.

## 2. Related Works

Many clustering methods have been proposed for WSNs, and the effectiveness of sensor nodes clustering for prolonging the network lifetime has been verified. In this section, existing methods for clustering sensor nodes are reviewed from two aspects, namely, non-computational intelligence and computational intelligence.

### 2.1. Clustering Sensor Nodes Based on Non-Computational Intelligence

Many methods for sensor node clustering based on non-computational intelligence have been proposed. These methods group sensor nodes into several clusters and select a CH for each cluster. The CH can gather data from other sensor nodes within the same cluster and send the aggregated data to the sink. This effectively saves energy and prolongs the network lifetime.

For clustering of sensor nodes based on non-computational intelligence, LEACH is a very popular clustering routing protocol for WSNs proposed by Heinzelman et al. [9]. In LEACH, some sensor nodes are randomly selected as CHs. Each CH aggregates data from the sensor nodes within its own cluster, and then sends the data to the sink. LEACH has the advantage of increasing energy efficiency, but, its problem is to randomly select cluster heads, sensor nodes with more residual energy and less residual energy have the same probability of being selected as CH. If sensor nodes with less residual energy are selected as CHs, then they die quickly. To solve this problem, a stable election protocol (SEP) for clustered heterogeneous WSNs is proposed by Smaragdakis et al. [29]. In SEP, a certain percentage of sensor nodes are equipped with more energy than other sensor nodes. The selection of CHs is based on the election weight according to the remaining energy of each node. However, neither LEACH nor SEP takes into account the location and distance information of sensor nodes. Therefore, a quadrant-based routing protocol (Q-LEACH) is proposed by Manzoor et al. [30]. In Q-LEACH, the sensing region is divided into four quadrants, and the area within each quadrant is regarded as a cluster. The CH of each cluster communicate with each other to determine the shortest route between the source and the destination. The Q-LEACH protocol prolonged the network lifetime while increased congestion in WSNs. Marappan and Rodrigues [31] proposed a cross layer-low energy adaptive clustering hierarchy model (CL-LEACH) to save the energy of sensor nodes. CL-LEACH considered the residual energy of sensor nodes for CH selection, which preserves the overall energy. In addition, some scholars proposed the grid-based sensor node clustering strategy [10,11,12]. For the grid-based clustering method, the sensing region is regularly divided into several subregions, and each subregion is regarded as a cluster, which can avoid the redundant data and long-distance communications. But the grid-based clustering strategies lead to an imbalance in the number of sensor nodes between clusters, thereby resulting in an imbalance in the energy consumption between CHs.

### 2.2. Clustering Sensor Nodes Based on Computational Intelligence

Clustering sensor nodes based on computational intelligence is another active research trend [32,33,34,35,36,37,38,39]. Compared with the non-computational intelligence methods, technologies of computational intelligence has the characteristics of self-learning, self-organization, and self-adaptive, which enables them to provide more effective solutions for sensor node clustering. The researches have tried to optimize the sensor node clustering with computational intelligence technologies from many different aspects. For example, Zhou et al. [32] optimized the cluster structure of sensor nodes to minimize the transmission distance and reduce the energy consumption of the WSNs by an improved PSO, in which the inertial weight of PSO is adjusted to avoid particles being trapped in local optima. To address the problem where the probability of selecting an optimal sensor node as rendezvous point are very low, Tabibi and Ghaffari [33] proposed a particle swarm optimization based selection (PSOBS) method to select the optimal rendezvous points which were responsible for gathering the data of other sensor nodes. To balance the load of CHs so as to prolong the network lifetime, Ray and De [34] proposed an energy efficient clustering protocol based on K-means algorithm (EECPK-means). The EECPK-means method can balance the number of sensor nodes between clusters. To maximize the lifetime of the network, Lata et al. [35] proposed a LEACH-Fuzzy clustering method which realized CH selection and cluster formation based on fuzzy logic. To effectively cluster the sensor nodes and select the optimal CH for each cluster, Fei et al. [36] proposed a hybrid clustering method based on fuzzy c means (FCM) and moth-flame optimization method (MFO) to improve the network quality (FCMMFO). In FCMMFO, FCM was used to form energy-efficient clusters and MFO was used to select the optimal CH for each cluster. To address uneven clustering and poor energy consumption, an inter-cluster multi-hop hierarchical routing algorithm based on iterative self-organizing data analysis techniques algorithm (IICMH) is proposed by Yang et al. [37]. In addition, Elhabyan et al. [38] proposed a single multi-objective problem to find the best network configuration formulation. The proposed formulation took into account the number of CHs, the number of clustered nodes, the link quality between the cluster members and CHs, and the link quality of the constructed routing tree. Furthermore, Wang et al. [39] proposed an improved artificial bee colony algorithm to select the CHs, which took into account the energy of CHs, the density of CHs, the location of CHs, and other similar factors.

The existing sensor node clustering methods based on computational intelligence have proved the effectiveness of computational intelligence technologies in providing effective solutions for WSNs. However, sensor nodes are usually randomly scattered in the sensing region. Most existing sensor node clustering methods do not balance the number of sensor nodes between clusters, which causes unbalanced energy consumption. In addition, in different applications, the distribution and number of sensor nodes are different, it is critical to automatically determine the number of clusters according to the number and distribution of sensor nodes. Furthermore, existing technologies of computational intelligence still have some shortcomings. For some optimization methods of computational intelligence, it is easy to converge into the local optimum when dealing with high-dimensional and complex problems. Additionally, in order to cope with different WSN problems, technologies of computational intelligence should be improved accordingly. To prolong the network lifetime and make WSN systems effective data collection [40], the sensor node clustering strategy and the performance of computational intelligence technologies need to be further studied.

Therefore, this work proposes an energy-balanced cluster-routing protocol using PSO with five mutation operators for WSN, which can automatically determine the number of clusters and balance the number of sensor nodes between clusters. In addition, five mutation operators are specially proposed to improve the performance of PSO in optimizing sensor node clustering by increasing the search diversity. The energy-balanced cluster-routing protocol can effectively balance the energy consumption of sensor nodes and thus prolonging the network lifetime.

## 3. Network Model

**Figure 2:**
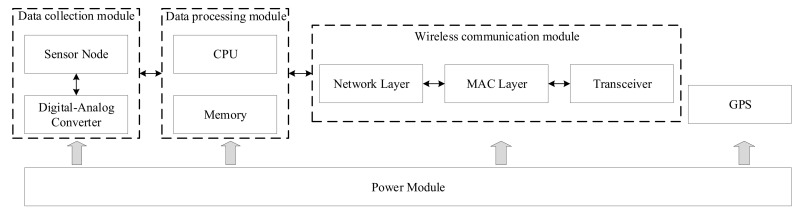
The main architecture of sensor nodes comprising four modules: data collection module, data processing module, wireless communication module, and power module.

In this study, the WSN system comprising *N* static sensor nodes and one sink node. The sink is placed in the center of the sensing region *R*, and sensor nodes are randomly scattered over *R* to monitor the environment. Sensor nodes can be grouped into several clusters, with one sensor node being appointed as the CH to aggregate data from the remaining cluster members within the same cluster. The set of sensor nodes is defined as $S=\{s_{1},s_{2},\ldots ,s_{N}\}$, and the set of CHs is defined as $\mathrm{CH}=\{ch_{1},ch_{2},\ldots ,ch_{k}\}$, where *k* is the number of clusters. Each CH creates a time division multiple access (TDMA) communication message for its cluster members, which is used to inform cluster members to send data to the corresponding CH according to the schedule. The data collection, processing, and communication functions of sensor nodes are determined by the architecture of the nodes (Figure 2).

Sensor nodes can convert their sensed information into digital signals in the data collection module. Then, the data processing module processes and stores these data. Sensor nodes can interact with each other via a wireless link within their communication range. The information they exchange includes collected data and some control information. Each Sensor node can locate its position through the global positioning system (GPS). The power module can provide energy to the above mentioned functional modules. Most of the available energy is consumed during data transmission. Unreasonable data transmission can lead to high and unbalanced energy consumption of sensor nodes, thereby reducing the lifetime of the network. The energy consumption model and network lifetime are described in the following subsections based on the following assumptions.

- All sensor nodes have the same communication range and equipment with the same initial energy.
- Sensor nodes can know their location via the GPS.
- The sink node has enough energy; the battery of sensor nodes cannot be replaced or recharged.
- Each sensor node has data of the same size to transmit in each round.

### 3.1. Energy Dissipation Model of Sensor Nodes

The energy consumed by sensor nodes is mainly used for data transmission. In this study, sensor nodes can be divided into two types: cluster members and CHs. Both of them can obtain data from the sensing region. Cluster members send the acquired data to their CH instead of directly to the sink. Each CH can aggregate data from its cluster members and send these data to the sink. The energy of cluster members is mainly used for sending data; the energy consumption of CHs includes both sending and receiving data. To calculate energy consumption, the channel model is employed based on the Euclidean distance *d* between the sender and the receiver. If *d* is smaller than a threshold $d_{0}$, the free channel model is selected; if $d>d_{0}$, the multipath model is adopted. The energy used by a sensor node to transmit or receive an *L*-bit packet is $E_{T}$ and $E_{R}$ respectively. $E_{T}$ can be calculated as Equation (1):

(1) $$\begin{array}{c} E_{T}=\left\{\begin{array}{ccc} L\times E_{e}+L\times E_{f}\times d^{2}, & & \;d<d_{0}\\ L\times E_{e}+L\times E_{m}\times d^{4}, & & \;d\geq d_{0}, \end{array}\right. \end{array}$$

where $E_{e}$ denotes the energy consumed to run the transmitter or receiver circuit; $E_{f}$ and $E_{m}$ depend on the used transmitter amplifier model (i.e., free channel or multipath model); $d_{0}$ denotes the threshold used to determine the model to be selected and can be defined as $\sqrt{E_{f}/E_{m}}$. $E_{R}$ is defined as Equation (2):

(2) $$\begin{array}{c} E_{R}=L\times E_{e}. \end{array}$$

It can be noticed from Equations (1) and (2) that the energy consumption of data transmission is greatly affected by the data size and distance between the sender and the receiver while the energy consumption of data reception is affected by the size of the received data. To reduce the energy consumption of sensor nodes and prolong the network lifetime, the data transmission distance and received data size should be reduced to lower the energy consumption of cluster members and CHs.

### 3.2. Lifetime Model of the Network

In this study, the lifetime of the network is considered to start from its initialization until it cannot work effectively. Generally, some existing studies define the network lifetime as a period from network initialization until the energy of at least one sensor node is depleted, that is, one sensor node fails [8,41,42]. The region covered by sensor nodes may not be monitored continuously after the energy of some sensor nodes is depleted, especially when a small number of sensor nodes are deployed in the sensed region; this situation can result in data loss. For a WSN system with a large number of sensor nodes, the network may continue to work effectively until a certain percentage of sensor nodes fail; this percentage is then used to determine the network lifetime [32].

Balancing the energy consumption of sensor nodes can extend the network lifetime of both small and large WSNs systems, because the energy consumption of sensor nodes become similar in this case. This can avoid the phenomenon where some sensor nodes consume energy faster while other sensors have a lot of residual energy, which is helpful to prolong the lifetime of WSNs.

## 4. EBCRP: Energy-Balanced Cluster-Routing Protocol

**Figure 3:**
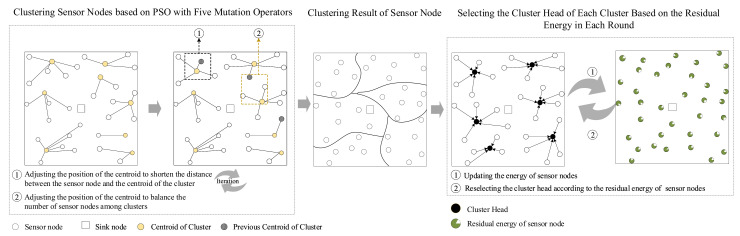
Main procedure of the proposed energy-balanced cluster-routing protocol (EBCRP).

Figure 3 shows the main procedure of the proposed EBCRP.

Based on the sensing region *R* with randomly deployed sensor nodes, the sensor nodes are first grouped into clusters evenly using PSO with five mutation operators. In this step, the numbers of clusters and their centroids are optimized, in which some sensor nodes are selected as the centroids of clusters and other sensor nodes are assigned to their respective nearest centroid. Additionally, the sensor node clustering scheme is optimized according to the following two objectives: minimizing the average distances between sensor nodes and their centroids, and maximizing the balance index of the number of sensor nodes between clusters. To increase the diversity of search, five mutation operators are proposed for optimizing the sensor node clustering scheme. Finally, the data collection task is performed based on the clustering results. In each round, the sensor node with the highest residual energy in each cluster is selected as a CH. Each CH is responsible for aggregating data from its cluster members and sending these data to the sink. The residual energy of all sensor nodes is also updated in each round until the end of the network lifetime.

### 4.1. Clustering Sensor Nodes Based on Particle Swarm Optimization with Five Mutation Operators

#### 4.1.1. Particle Swarm Optimization

PSO is a population-based global optimization technology; the individuals in the population are called particles. In PSO, a swarm uses *M* particles to search for the best solution, with each particle representing a potential solution. Particles have two attributes: velocity *v* and position *x*. The solution (position) of the *i*-th particle can be expressed as $x_{i}=\left\{x_{i1},x_{i2},\ldots ,x_{iD}\right\}$, where *D* is the dimension of particles. The velocity of the *i*-th particle, $v_{i}=\left\{v_{i1},v_{i2},\ldots ,v_{iD}\right\}$, represents the speed of particle movement. In PSO, particles can cooperate and share information to find the best solution. All particles iteratively adjust their velocity and position according to their best experience Pbest and the best experience Gbest of the entire swarm. The scholars added the inertia weight *w* to the traditional PSO to change the convergence speed of the optimization algorithm, which is called standard PSO [43]. The velocity and position of the *i*-th particle are updated by Equations (3) and (4), respectively.

(3) $$\begin{array}{ccc} v_{i} & = & wv_{i}\;+\;rc_{1}\left(Pbest_{i}\;-\;x_{i}\right)\;+\;rc_{2}\left(Gbest\;-\;x_{i}\right), \end{array}$$

(4) $$\begin{array}{ccc} x_{i} & = & x_{i}+v_{i}, \end{array}$$

where *w* represents the inertia weight, $c_{1}$ and $c_{2}$ are acceleration constants, *r* is a random value in [0,1], and $Pbest_{i}$ is the best experience of the *i*-th particle.

#### 4.1.2. Topology of Particles in Sensor Node Clustering

**Figure 4:**
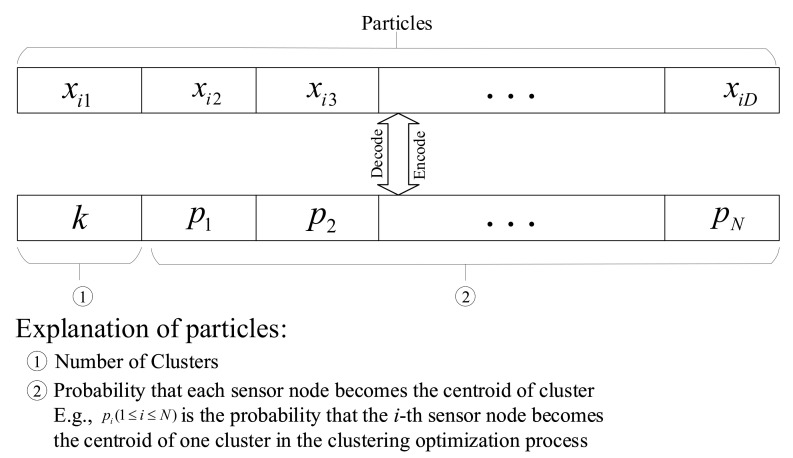
Structure of particles utilized in sensor node clustering optimization.

In EBCRP, PSO is used to optimize the number of clusters and centroid of each cluster. Figure 4 illustrates the structure of particles utilized in sensor node clustering optimization.

A total of D=N+1-dimensional parameters are optimized, where *N* denotes the number of sensor nodes. In this structure of particles, the first dimension represents the number of clusters *k*, while the *i*-th (i∈{2,…,N+1}) dimension represents the probability $P_{i}\in \left[0,1\right]$ that the (i−1)-th sensor node is selected as the centroid of a cluster.

During the optimization process, for each particle (solution), *k* sensor nodes with the highest probability are selected as cluster centroids of clusters according to the first dimension parameter *k* in the particle. Other sensor nodes are assigned to their nearest centroid, which forms the sensor node clustering solution (scheme) of each particle in each generation.

#### 4.1.3. Cost Function in Sensor Node Clustering

Each particle corresponds to a sensor node clustering solution in each generation. These solutions are expected to be searched towards the desired target (i.e., shorting the distance between sensor nodes and their centroids while balancing the number of sensor nodes between clusters). Therefore, the fitness of the clustering solutions need to be evaluated.

In this study, two goals are expected. Firstly, it is hoped that the nearest sensor nodes are grouped into a cluster instead of any distance. The average distance between the sensor nodes and the centroids they belong to is calculated to achieve the goal, which is expected to have a small value. Secondly, balancing the number of sensor nodes between clusters is expected, which helps to balance energy consumption of sensor nodes so as to prolong the lifetime of WSNs. Jain’s fairness index [44] is used to evaluate the balance index of the number of sensor nodes between clusters. The corresponding balance index can be defined as Equation (5):

(5) $$BI=\frac{{\left(\sum_{i=1}^{k}n_{i}\right)}^{2}}{k(\sum_{i=1}^{k}n_{i}^{2})},$$

where BI is the balance index of the number of sensor nodes between clusters, $n_{i}$ is the number of sensor nodes in the *i*-th cluster. The value of BI ranges in the interval of [0, 1]. When BI is close to 1, the more balanced the number of sensor nodes between clusters. Therefore, the optimization objective can be defined to maximize the Equation (6):

(6) $$max\;\;fit=\frac{w_{ad}}{Sum_{d}/\left(\sum_{i=1}^{k}n_{i}\right)}+BI,$$

where $Sum_{d}$ represents the sum of the distances between all sensor nodes and their centroids, $\sum_{i=1}^{k}n_{i}$ means the number of sensor nodes in all clusters, that is, $\sum_{i=1}^{k}n_{i}=N$, $Sum_{d}/N$ represents the average distance, and $w_{ad}$ is the weight of the average distance in fitness fit ($w_{ad}=10$). Since *N* is a positive integer, Equation (6) is equivalent to Equation (7).

(7) $$max\;\;fit=\frac{10}{Sum_{d}}+\frac{N}{k(\sum_{i=1}^{k}n_{i}^{2})}.$$

#### 4.1.4. Five Mutation Operators

In the sensor node clustering optimization process, *k* (the value of the first dimension of a particle) sensor nodes with the highest probability are selected as cluster centroids of clusters. This means that it is necessary to not only simply optimize the value of each dimension of particles, but also adjust the position (dimension) of the value corresponding to the highest *k* probabilities. In addition, the clustering solution is significantly affected by the first dimension of particles representing the number of clusters. To increase the search diversity, the following five mutation operators are proposed for optimizing the sensor node clustering scheme.

- Reversing probability operator: it is used to reverse a part of the probabilities of sensor nodes. The detail process is shown in Algorithm 1.
- Decreasing probability operator: it is used to decrease the probability of some sensor nodes becoming cluster centroids. The detail process is shown in Algorithm 2.
- Increasing probability operator: it is used to increase the probability of some sensor nodes becoming the cluster centroids. The detail process is shown in Algorithm 3.
- Swapping probability operator: it is used to swap the probabilities of two sensor nodes in a particle. The detail process is shown in Algorithm 4.
- Transforming number operator: it is used to transform the number of clusters in the first dimension of particles. The detail process is shown in Algorithm 5.

**Algorithm 1:** Reversing probability operator

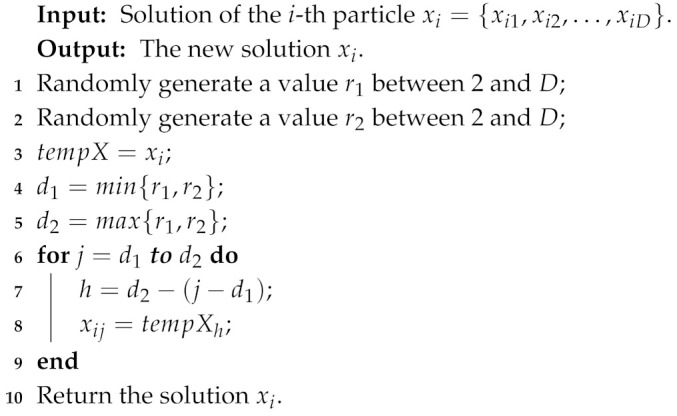



**Algorithm 2:** Decreasing probability operator

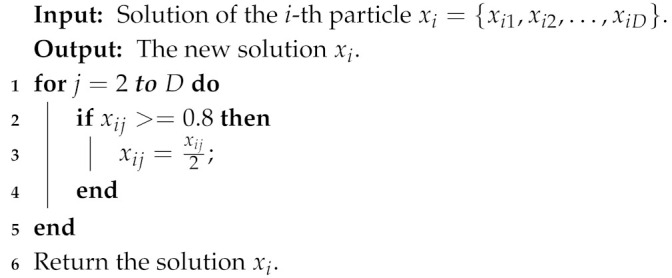



**Algorithm 3:** Increasing probability operator

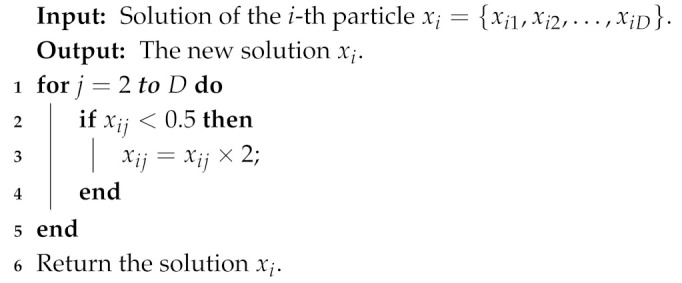



**Algorithm 4:** Swapping probability operator

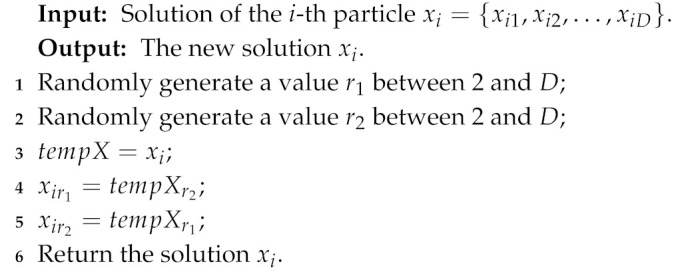



**Algorithm 5:** Transforming number operator

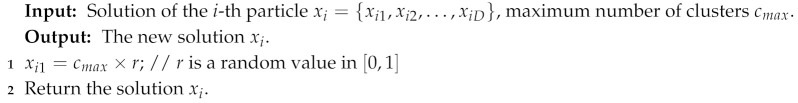



#### 4.1.5. Optimization Process of Sensor Node Clustering

This section outlines the process of clustering sensor nodes based on PSO with the introduced five mutation operators. The parameters of PSO (position *x*, velocity *v*, and fitness) are first initialized for all particles. The best experience of particles (Pbest) and the best experience of entire swarm (Gbest) are also initialized. Then, particles begin to search for the best sensor node clustering solution through iterations. In each iteration, each particle updates its velocity and position; each particle also mutates when a random value r>0.5 that is used to judge whether the current *i*-th particle is mutated. During the mutation process, five mutation operators are randomly selected to apply to the position for the *i*-th particle. Next, the fitness $fit_{i}$ is evaluate. The best experiences $Pbest_{i}$ and Gbest are updated if $fit_{i}$ is higher than them. Until the stopping criterion is satisfied, the best clustering solution is obtained. The entire optimization process is shown in Algorithm 6.    
**Algorithm 6:** Clustering sensor nodes based on PSO with five mutation operators
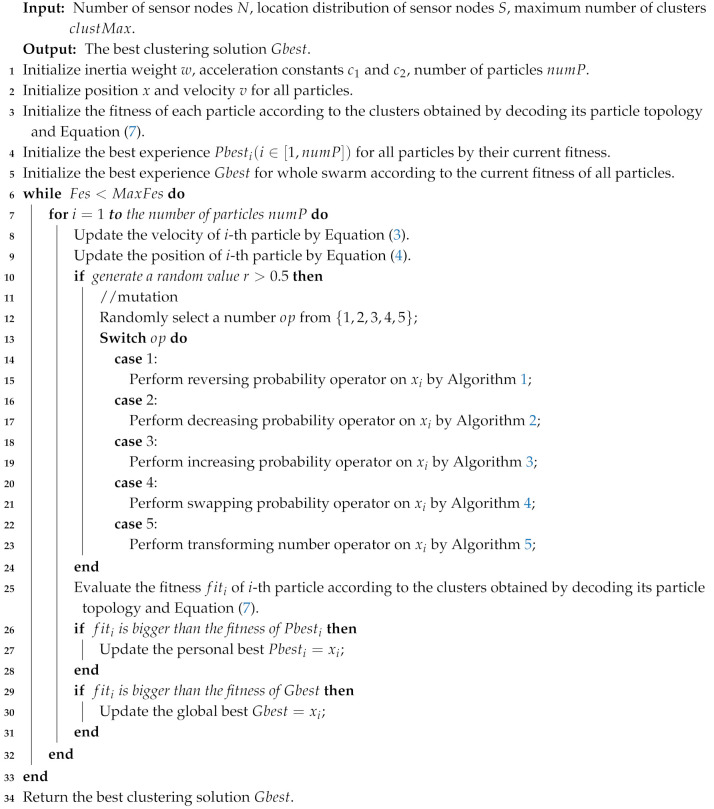


### 4.2. Selecting the Cluster Heads Based on Residual Energy

Sensor nodes are grouped into *k* clusters based on PSO with five mutation operators as described in the above subsection, in which the number of clusters *k* is optimized by PSO. The set of clusters CS is defined as $\mathrm{CS}=\{cs_{1},cs_{2},\ldots ,cs_{k}\}$. $cs_{i}$ has a set of sensor nodes, $cs_{i}=\left\{s_{i}^{1},\ldots ,s_{i}^{n_{i}}\right\}$, where $s_{i}^{j}$ denotes the *j*-th sensor node in $cs_{i}$ and $n_{i}$ is the number of sensor nodes in $cs_{i}$.

In this study, a rotation CH selection scheme based on the highest residual energy is used to balance the energy consumption of sensor nodes, thereby prolonging the network lifetime. In each round, the sensor node with the highest residual energy in each cluster is selected as a CH to aggregate data from its cluster members and send data to the sink. During the lifetime of the network system, the residual energy of sensor nodes in $cs_{i}$ is defined as $e_{i}=\left\{e_{i}^{1},\ldots ,e_{i}^{n_{i}}\right\}$. The set of CHs in each round is defined as $\mathrm{CH}=\{ch_{1},ch_{2},\ldots ,ch_{k}\}$ and calculated as Equation (8).

(8) $$ch_{i}=\left\{j\in \left[1,n_{i}\right]\;|\;e_{i}^{j}\geq e_{i}^{k},k\in \left[1,n_{i}\right]\right\}.$$

## 5. Simulations and Results

This section presents several simulations performed using MATALAB to evaluate the effectiveness and performance of the proposed EBCRP method. The effectiveness of EBCRP is first validated in terms of the clustering performance and balanced energy consumption of sensor nodes. Further, simulations are also performed to compare EBCRP with some existing methods, namely, LEACH [9], SEP [29], and IICMH [37] in terms of the network lifetime and balancing the energy consumption. For the simulations, the sensing region is set to 100 m × 100 m; the number of sensor nodes *N* is varied from 50 to 200. Some parameters of the WSN system are listed in Table 1.

### 5.1. The Effectiveness of EBCRP

In EBCRP, an adaptive sensor node clustering scheme based on PSO is proposed to determine the number of clusters and group sensor nodes into clusters evenly. Additionally, five mutation operators are specially proposed to improve the performance of PSO in optimizing sensor node clustering. This subsection examines the effectiveness of the proposed EBCRP method.

**Figure 5:**
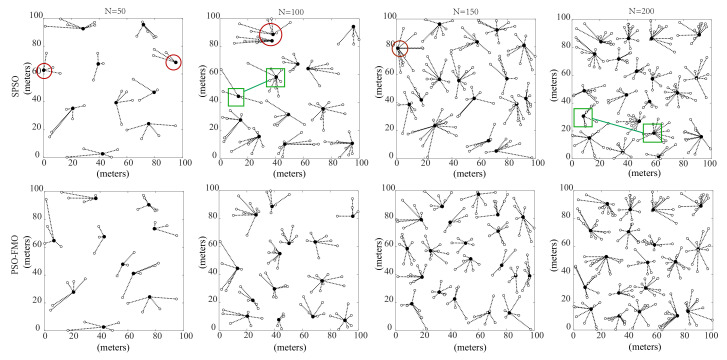
Clustering results of sensor nodes based on SPSO and PSO-FMO in terms of different number of sensor nodes 50, 100, 150 and 200. The clusters highlighted by circles have extremely asymmetrical centroids; while the clusters highlighted by square show the imbalance of the number of sensor nodes between clusters, which is the result of SPSO.

The effectiveness of PSO and five mutation operators in EBCRP are verified. PSO with five mutation operators (PSO-FMO) and without five mutation operators (SPSO) are compared. The same parameters are used for both PSO-FMO and SPSO. The population size is set to 50. Acceleration constants $c_{1}$ and $c_{2}$ are set to 1.5 and 2.0, respectively. The number of fitness evaluations (FEs) is set to 10,000. Figure 5 shows the sensor node clustering results based on SPSO and PSO-FMO for different numbers of sensor nodes (50, 100, 150 and 200).

It can be noticed from the figure that while both PSO-FMO and SPSO group the sensor nodes scattered into clusters in the sensing region *R*. However, the allocation of cluster centroids by SPSO is not reasonable. For example, the clusters highlighted by circles have extremely asymmetrical centroids, which results in an increased distance between sensor nodes and their centroids and thus a deceased fitness fit. Furthermore, the result of SPSO also shows the imbalance of the number of sensor nodes between clusters, which is shown in the clusters highlighted by square. These results indicate the weak performance of SPSO, the method that does not use the five mutation operators in optimizing sensor node clustering. In contrast, PSO-FMO achieves more reasonable cluster centroids and a better balance of the number of sensor nodes between clusters. This is because the use of five mutation operators increases the search diversity, thus improving the probability of finding a better sensor node clustering solution. Therefore, the use of PSO-FMO justifies the good performance of EBCRP in optimizing sensor node clustering.

**Figure 6:**
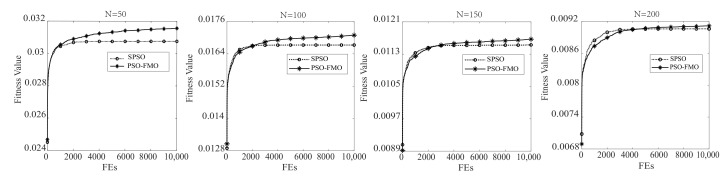
Mean convergence curves of SPSO and PSO-FMO in optimizing sensor node clustering.

Figure 6 illustrates the mean convergence curves of SPSO and PSO-FMO in optimizing sensor nodes clustering.

To reduce the statistical error, both methods are run 30 times under the same conditions. It can be noticed from the figure that PSO-FMO achieves a higher average fitness for different numbers of sensor nodes compared to SPSO. SPSO converges quickly and falls into a local optima. In contrast, PSO-FMO continuously searches the best solution and avoids falling into local optima. This is because the five mutation operators continuously provide the diversity of particles for the entire swarm, which helps particles find a better solution. Hence, the mean convergence curves also verify the effectiveness of PSO-FMO in EBCRP.

To verify the effectiveness of PSO-FMO in balancing the number of sensor nodes between clusters, Table 2 also gives the comparison of balance index of the number of sensor nodes between clusters. It can be noticed from the table that PSO-FMO achieves a better balance for different numbers of sensor nodes, which also indicates the effectiveness of PSO-FMO in EBCRP.

**Figure 7:**
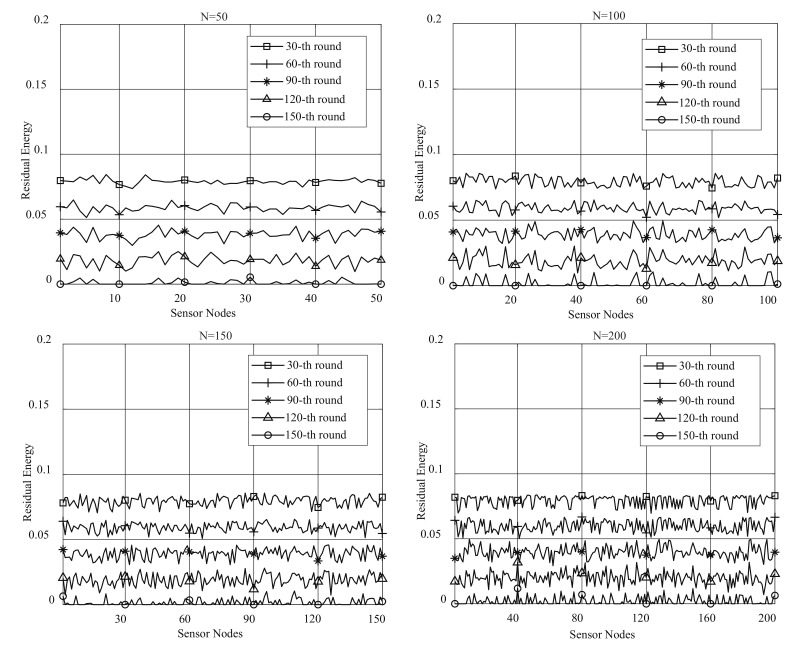
Residual energies of sensor nodes in different rounds.

To verify the effectiveness of EBCRP method in balancing the energy consumption of sensor nodes, the residual energy of sensor nodes during the network lifetime is analyzed. Figure 7 shows the residual energy of each sensor node in different rounds for different numbers of sensor nodes (50, 100, 150 and 200).

While the residual energies of sensor nodes fluctuate up and down, it still demonstrates balance within a small energy range. In sensor node clusters, some sensor nodes are dynamically selected as CHs, while others are selected as cluster members. The rates of energy consumption of CHs and cluster members are different. Therefore, there are some sensor nodes with a higher energy consumption. Furthermore, all sensor nodes cannot consume energy completely synchronously. Small range fluctuations of the residual energy of sensor nodes prove the effectiveness of EBCRP in balancing the energy consumption of sensor nodes.

### 5.2. Comparison of EBCRP with Other Methods

To validate the performance of the proposed EBCRP method, it was compared to other methods, namely, LEACH [9], SEP [29], and IICMH [37]. The LEACH protocol selects some sensor nodes as CHs according to a random probability of each node. Other sensor nodes were assigned to corresponding CHs based on the received signal strength. SEP is similar to LEACH, except that a certain proportion of sensor nodes in SEP are equipped with more energy, and CHs are selected based on the residual energy of each sensor node. IICMH adopts iterative self-organizing data analysis techniques algorithm to select clustering centers; then, CHs are selected based on the distance between sensor nodes and the clustering centers. When the residual energy of CHs is lower than a set threshold, CHs are reselected based on the residual energy of sensor nodes.

To fully compare the performance of each method, simulations were performed for four different numbers of sensor nodes: 50, 100, 150 and 200. For LEACH and SEP, 5% of sensor nodes were selected as CHs. For the IICMH and EBCRP methods, 5% of sensor nodes are set as the expected number of clusters and a maximum number of clusters, respectively.

**Figure 8:**
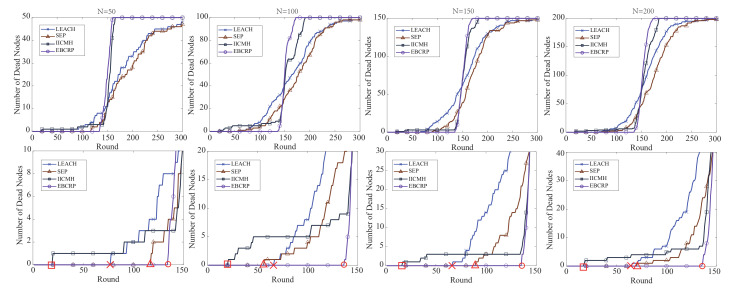
Number of dead nodes of different methods in different rounds, and the bottom subfigures detail the the first dead node.

Figure 8 shows the number of dead sensor nodes for the different methods in different rounds.

The first row displays the number of dead sensor nodes from 0 to 300 rounds, while the second row shows the round corresponding to the first dead sensor node for each method. It can be noticed from the figure that the number of dead sensor nodes in LEACH and SEP increases slowly. It is obvious that some sensor nodes consume energy too fast, while others consume energy slowly due to the unbalanced energy consumption of sensor nodes in LEACH and SEP. In contrast, IICMH considers the balance of the number of sensor nodes between clusters when clustering sensor nodes, which helps balance the energy consumption of sensor nodes. Therefore, there is a period of a sharp increase in the number of dead sensor nodes. Nevertheless, IICMH still does not deal with the energy balance of sensor nodes well as some sensor nodes ran out of energy prematurely. EBCRP only shows a period of a sharp rise in the number of dead sensor nodes, whereas there is no problem of sensor nodes consuming energy prematurely. This is because EBCRP balances the energy consumption of sensor nodes well by balancing the number of sensor nodes between clusters and employing the rotation CH selection scheme based on the highest residual energy. It can be noticed that the first dead sensor node appears in a later round for EBCRP compared to the other methods, which also indicates that the energy balance of sensor nodes in EBCRP is better. Overall, EBCRP presents a good performance in balancing the energy consumption of sensor nodes.

**Figure 9:**
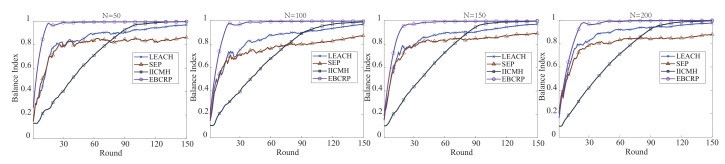
The curve of energy consumption balance index of different methods.

To further verify the balance of energy consumption between sensor nodes in EBCRP, the energy consumption balance index (ECBI) is calculated based on the fairness index as shown in Equation (9).

(9) $$ECBI=\frac{{\left(\sum_{i=1}^{N}ec_{i}\right)}^{2}}{N(\sum_{i=1}^{N}ec_{i}^{2})},$$

where $ec_{i}$ is the energy consumption of the *i*-th sensor node. Figure 9 compares the curve of ECBI of different methods during rounds.

In addition, Table 3 also quantitatively compares the ECBI between the proposed EBCRP method and other methods in 30-th, 60-th, 90-th, 120-th, and 150-th rounds under the different number of sensor nodes (50, 100, 150 and 200), in which the best EBCI value is bolded for each round.

From Figure 9 and Table 3, it can be observed that the ECBI of all the methods increases gradually with the increase of rounds from 1 to 150 for all the case with different numbers of sensor nodes. This is because more and more sensor nodes consume a lot or even all energy with the increase of rounds, which will raise the ECBI. Also, the proposed EBCRP method and these comparison methods dynamically select CH for each cluster, which can balance the energy consumption between sensor nodes. However, the ECBI of the proposed EBCRP method is significantly higher than that of the other methods in different rounds. This is because LEACH and SEP randomly select some sensor nodes as CHs, and then other sensor nodes are assigned to their respective nearest CHs, which ignores the balance of the number of sensor nodes between clusters and increases the imbalance of energy consumption between sensor nodes. In contrast, during sensor node clustering, IICMH and the proposed EBCRP take into account the balance of the number of sensor nodes between clusters, thereby helping to balance the energy consumption of sensor nodes. Nevertheless, in the IICMH method, the CH of each cluster is reselected after the energy of the previous CH is lower than a threshold. This makes the energy of sensor nodes selected as CHs for the first time consume more energy. On the contrary, the used rotation CH selection based on the highest residual energy in the proposed EBCRP method can effectively balance the energy consumption between sensor nodes. So the ECBI of IICMH is lower than that of the proposed EBCRP method. The significantly higher ECBI of the proposed EBCRP than other methods prove the effectiveness of EBCRP in balancing the energy consumption between sensor nodes. Furthermore, EBCRP shows a high ECBI in different rounds, whereas the other methods show significantly smaller ECBI in the early rounds than in the later rounds. This also proves the advantages of EBCRP over other methods. Therefore, the proposed EBCRP performs well in balancing the energy consumption of sensor nodes. However, the proposed EBCRP method dynamically selects CH for each cluster in each round, which reduces the stability of the network system.

**Figure 10:**
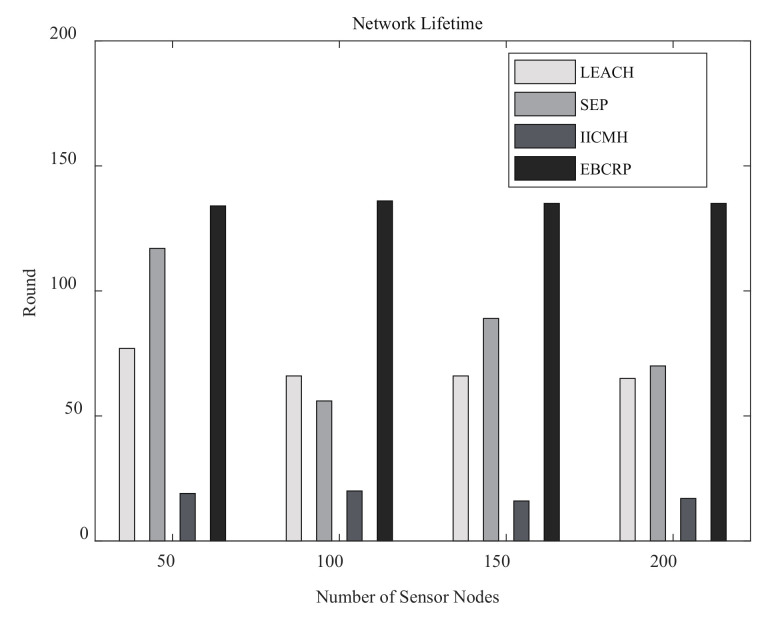
Network lifetime of different methods for different numbers of sensor nodes.

The network lifetime of EBCRP is also compared with other methods. Figure 10 illustrates the network lifetime of different methods for different numbers of sensor nodes.

EBCRP achieves the longest lifetime compared to LEACH, SEP, and IICMH. There are two main factors that prolong the network lifetime of EBCRP. First, EBCRP employs an adaptive sensor node clustering scheme to determine the number of clusters and group sensor nodes into clusters evenly. This helps to balance the energy consumption between clusters. Second, EBCRP adopts a rotation CH selection scheme based on the highest residual energy to dynamically select CHs for each cluster. This approach can balance the energy consumption of sensor nodes within each cluster. Balancing the energy consumption between clusters and within clusters in EBCRP prevents some sensor nodes from consuming energy prematurely, thereby prolonging the lifetime of the WSN. However, LEACH and SEP randomly select some sensor nodes as CHs, while other sensor nodes are assigned to their respective nearest CHs, which cannot balance the number of sensor nodes between clusters and energy consumption of sensor nodes. At the same time, a certain proportion of sensor nodes in SEP are equipped with more energy, and CHs selection in SEP takes into account the residual energy of sensor nodes. Thus, the network lifetime of SEP is higher than that of LEACH, but it is still lower than the proposed EBCRP method. Although the IICMH also considers the balance of the number of sensor nodes between clusters, the CH of each cluster is reselected after the energy of the previous CH is lower than a threshold. This makes the energy of sensor nodes selected as CHs for the first time to quickly depleted. In addition, sensor nodes close to the sink in each cluster are preferentially selected as CHs in IICMH, which increases the energy consumption of some sensor nodes (i.e., cluster members far away from their CHs) when they send data to their CHs. Thus, the network lifetime of IICMH is lower compared to the other methods. Overall, the proposed EBCRP method performs better in prolonging the network lifetime by performing balanced clustering and employing the rotation CH selection scheme based on the highest residual energy.

## 6. Conclusions

This study proposes an energy balanced cluster-routing protocol based on particle swarm optimization with five mutation operators for wireless sensor networks to balance energy consumption of sensor nodes and prolong the network lifetime. An adaptive sensor node clustering scheme based on the PSO is proposed to determine the number of clusters and group sensor nodes into clusters evenly, which is helpful to balance the energy consumption between sensor nodes. To increase the search diversity, five mutation operators are specially proposed in EBCRP to improve the performance of PSO in optimizing sensor node clustering. In addition, a rotation CH selection scheme based on the highest residual energy is used in EBCRP to dynamically select a CH for each cluster. In each round, the sensor node with the highest residual energy in each cluster is selected as a CH responsible for aggregating data from the remaining sensor nodes within each cluster and sending these data to the sink. This can balance the energy consumption of sensor nodes within each cluster. Comprehensive simulations are performed to investigate the effectiveness of the proposed EBCRP method and compare it to some other methods. The simulation results demonstrate that the proposed EBCRP method can effectively balance the number of sensor nodes between clusters and energy consumption of sensor nodes, thereby prolonging the network lifetime.

Some limitations still exist in the EBCRP method and become important research work in the future. The PSO-based sensor node clustering scheme is greatly affected by the performance of the PSO. For the multimodal, high-dimensional, complex, and nonlinear optimization problems in WSNs, the PSO is easy to prematurely converges to local optima. To find an optimal or near-optimal solutions, PSO and other optimization algorithms could be studied in the future to further improve the performance of EBCRP. In addition, the EBCRP selects CH for each cluster in each round, which reduces the stability of the network system. A more efficient CH selection mechanism could be further studied, e.g, each cluster selects multiple CHs to cooperate with each other to aggregate the data from other sensor nodes within their cluster. When the energy of the current CHs is lower than a threshold, the new CHs are reselected. Another important future work is about the application of the EBCRP to complex WSNs problems with large sensing region, some relay nodes can be selected to assist the communication between CHs. 

## Figures and Tables

**Table 1:** Parameters and their values of the wireless sensor network (WSN).

Parameter	Value
Initial Energy of Sensor Nodes ($E_{0}$)	0.1 J
Size of Data	4000 bit
$E_{e}$	50 nJ/bit
$E_{f}$	10 pJ/bit${}^{2}$
$E_{m}$	1.3 × 10${}^{-3}$ pJ/bit${}^{4}$

**Table 2:** Balance Index of the Number of Sensor Nodes between Clusters.

Total Number of Sensor Nodes	N=50	N=100	N=150	N=200
Balance index based on SPSO	0.9615	0.9568	0.9527	0.9542
Balance index based on PSO-FMO	0.9766	0.9731	0.9715	0.9747

**Table 3:** The comparison of energy consumption balance index of different methods.

Number of Sensor Nodes	Round	LEACH	SEP	IICMH	EBCRP
	30-th	0.7883	0.7918	0.4026	**0.9906**
	60-th	0.8939	0.8362	0.7096	**0.9954**
*N* = 50	90-th	0.9056	0.8485	0.9282	**0.9967**
	120-th	0.9459	0.8270	0.9899	**0.9976**
	150-th	0.9712	0.8645	0.9992	**0.9997**
	30-th	0.7530	0.7094	0.3935	**0.9691**
	60-th	0.8784	0.7623	0.6768	**0.9937**
*N* = 100	90-th	0.8956	0.8005	0.8010	**0.9956**
	120-th	0.9346	0.8270	0.9712	**0.9965**
	150-th	0.9692	0.8719	0.9911	**0.9980**
	30-th	0.7894	0.7861	0.4364	**0.9722**
	60-th	0.8951	0.8276	0.7299	**0.9934**
*N* = 150	90-th	0.9042	0.8478	0.9464	**0.9960**
	120-th	0.9470	0.8683	0.9897	**0.9970**
	150-th	0.9725	0.8919	0.9973	**0.9994**
	30-th	0.8251	0.7858	0.4193	**0.9650**
	60-th	0.9185	0.8322	0.7107	**0.9890**
*N* = 200	90-th	0.9384	0.8402	0.9219	**0.9948**
	120-th	0.9584	0.8479	0.9843	**0.9966**
	150-th	0.9758	0.8785	0.9951	**0.9991**

The best results are highlighted in bold.
